# Semi-supervised discovery of differential genes

**DOI:** 10.1186/1471-2105-7-414

**Published:** 2006-09-18

**Authors:** Shigeyuki Oba, Shin lshii

**Affiliations:** 1 Graduate School of Information Science, Nara Institute of Science and Technology, Takayama, Ikoma, Nara, Japan

## Abstract

**Background:**

Various statistical scores have been proposed for evaluating the significance of genes that may exhibit differential expression between two or more controlled conditions. However, in many clinical studies to detect clinical marker genes for example, the conditions have not necessarily been controlled well, thus condition labels are sometimes hard to obtain due to physical, financial, and time costs. In such a situation, we can consider an unsupervised case where labels are not available or a semi-supervised case where labels are available for a part of the whole sample set, rather than a well-studied supervised case where all samples have their labels.

**Results:**

We assume a latent variable model for the expression of active genes and apply the optimal discovery procedure (ODP) proposed by Storey (2005) to the model. Our latent variable model allows gene significance scores to be applied to unsupervised and semi-supervised cases. The ODP framework improves detectability by sharing the estimated parameters of null and alternative models of multiple tests over multiple genes. A theoretical consideration leads to two different interpretations of the latent variable, i.e., it only implicitly affects the alternative model through the model parameters, or it is explicitly included in the alternative model, so that the interpretations correspond to two different implementations of ODP. By comparing the two implementations through experiments with simulation data, we have found that sharing the latent variable estimation is effective for increasing the detectability of truly active genes. We also show that the unsupervised and semi-supervised rating of genes, which takes into account the samples without condition labels, can improve detection of active genes in real gene discovery problems.

**Conclusion:**

The experimental results indicate that the ODP framework is effective for hypotheses including latent variables and is further improved by sharing the estimations of hidden variables over multiple tests.

## Background

Selecting significantly differential genes is one of the most important tasks in studies of gene expression analysis. This, however, is not an easy task because gene expression measurement studies include some difficulties, such as high noise, a small number of measurements (samples) in each condition, severe multiplicity of tests corresponding to a large number of genes, and so on. Our purpose is to improve the gene significance ranking score, by which a larger proportion of truly active genes is referred to as significant, while a greater proportion of truly inactive genes is called insignificant. Here, a gene is called "active" if its mean expression level has a relationship to the biological conditions of interest, and it is "inactive" if there is no such relationship.

Most simply, the significance of genes can be evaluated using p-values with the Bonferroni correction [1] corresponding to signal-to-noise ratios. Furthermore, there have been many studies undertaken to improve the detectability of active genes and/or evaluate the detected set of genes. Some of them have proposed problem-specific devices for microarray studies, and several researches have included new general methodologies for handling the multiplicity of statistical tests. Among them, the two most important ideas are (a) considering family-wise errors, and (b) sharing commonality among multiple tests. These ideas are not limited to microarray studies, but can also be applied to current and future bioinformatics subjects. Family-wise errors, such as the false discovery rate (FDR) [2], have become one of the most important ideas in data analysis of bioinformatics subjects involving severe multiplicity. It is becoming clearer that we cannot discuss the significance of each objective gene based on microarray studies; rather, we should handle a set of hypotheses by controlling the family-wise errors.

Sharing commonality among multiple tests has become another important issue in recent years, in order to increase the detectability from expressed genes that are highly correlated. The significance analysis of microarray (SAM) [3] considers alternative statistics of the signal-to-ratio statistics, *d*/(*S *+ *S*_0_), where *d *is a difference of class averages, *S *is a common standard deviation in each class for each gene, and *S*_0 _is an arbitrary positive constant appropriately given by the user. As for determining the value of *S*_0_, which stabilizes the signal-to-noise ratio statistics when *S *is too small, [4] proposed a criterion that *S*_0 _is set at the 5% point of *S *of all genes. It was the first and simplest way to share commonality among multiple tests. The empirical Bayes score [5] assumes a hierarchical Bayes model in which the priors of the parameters are common among all genes, and the priors are hierarchically estimated by using the expression of all genes. In the empirical Bayes model, the idea of sharing commonality appears more formally, which also explains the reason why SAM achieved similar improvement.

Very recently, Storey et al. [6,7] proposed a new framework, called the optimal discovery procedure (ODP), by extending Neyman-Pearson's lemma to multiple tests. The ODP considers direct optimization of a family-wise error by incorporating the idea of sharing commonality. Namely, the above two ideas (a) and (b) are strongly integrated in the ODP. Their ODP methodology is implemented in their point-and-click application software, EDGE [8].

In this study, we extend the ODP framework to general problems in which alternative distributions are defined by a latent variable model. Specifically, we propose a mixture model, a typical example of a hidden variable model, which assumes a stochastic noise process in each condition and includes a latent (hidden) variable indicating a class label of the condition to which the sample belongs. Since the label is regarded as a hidden variable, it is not necessarily provided. This model is then lent to two new significance scores of genes; an unsupervised significance score that assumes the class labels are not provided, and a semi-supervised significance score that can deal with samples either with or without class labels. We found that there are generally two theoretically natural but different ways to deal with the hidden variables in the ODP manner; namely, the estimated values of hidden variables are shared among multiple tests explicitly or implicitly through the model parameters. We compare them through a simulation experiment and show that explicit sharing of the commonality in the hidden variable improves the detectability of active genes. Using artificial and real data sets, we also show that the unsupervised and semi-supervised score of gene significance can improve the stability of active gene ranking against a small number of labeled samples by incorporating the unlabeled samples.

### Neyman-Pearson's lemma and the supervised gene significance score

Our objective is to accurately determine whether a gene *i *is active or inactive, according to the expression data *x*_*i *_= (*x*_*i*1_, *x*_*i*2_, ..., *x*_*iM*_) and label information *Y *= (*y*_1_, ...,*y*_*M*_), *y*_*j *_∈ {1, 2}, *j *= 1, ..., *M*, over *M *measurements (samples).

Following the conventional framework of statistical testing, let the null hypothesis that the gene is actually inactive be denoted as *H*_0 _, and the alternative hypothesis that the gene is actually active be denoted as *H*_1_. Our question is what kind of significance score *S *is best for discriminating active genes from inactive ones based on a limited number of measurements such as in conventional microarray experiments. The null and the alternative models can be represented as null and alternative probability density functions (PDFs), *f *(*X*_*i*_) = *p*(*X*_*i*_*|H*_0_) and *g*(*X*_*i*_) = *p*(*X*_*i*_*|H*_1_), respectively; namely, they are simple hypotheses with no variable parameter. Then, the following likelihood ratio score is known as the most powerful score of significance:

SLR(Xi)=g(Xi)f(Xi),     (1)
 MathType@MTEF@5@5@+=feaafiart1ev1aaatCvAUfKttLearuWrP9MDH5MBPbIqV92AaeXatLxBI9gBaebbnrfifHhDYfgasaacH8akY=wiFfYdH8Gipec8Eeeu0xXdbba9frFj0=OqFfea0dXdd9vqai=hGuQ8kuc9pgc9s8qqaq=dirpe0xb9q8qiLsFr0=vr0=vr0dc8meaabaqaciaacaGaaeqabaqabeGadaaakeaacqWGtbWudaWgaaWcbaGaeeitaWKaeeOuaifabeaakiabcIcaOiabdIfaynaaBaaaleaacqWGPbqAaeqaaOGaeiykaKIaeyypa0ZaaSaaaeaacqWGNbWzcqGGOaakcqWGybawdaWgaaWcbaGaemyAaKgabeaakiabcMcaPaqaaiabdAgaMjabcIcaOiabdIfaynaaBaaaleaacqWGPbqAaeqaaOGaeiykaKcaaiabcYcaSiaaxMaacaWLjaWaaeWaceaacqaIXaqmaiaawIcacaGLPaaaaaa@4630@

which was stated and proven as Neyman-Pearson's lemma [9].

Many useful statistical tests, however, assume non-simple hypothetical models so that the null and alternative PDFs include variable parameters to be estimated statistically. For example, in a typical approach for supervised differential gene discovery [10], the null and the alternative models are defined as

H0:f(Xi;φi)=∏jN(xij|0,σ0i2),     (2)
 MathType@MTEF@5@5@+=feaafiart1ev1aaatCvAUfKttLearuWrP9MDH5MBPbIqV92AaeXatLxBI9gBaebbnrfifHhDYfgasaacH8akY=wiFfYdH8Gipec8Eeeu0xXdbba9frFj0=OqFfea0dXdd9vqai=hGuQ8kuc9pgc9s8qqaq=dirpe0xb9q8qiLsFr0=vr0=vr0dc8meaabaqaciaacaGaaeqabaqabeGadaaakeaacqWGibasdaWgaaWcbaGaeGimaadabeaakiabcQda6iabdAgaMjabcIcaOiabdIfaynaaBaaaleaacqWGPbqAaeqaaOGaei4oaSdcciGae8NXdy2aaSbaaSqaaiabdMgaPbqabaGccqGGPaqkcqGH9aqpdaqeqbqaaiabd6eaojabcIcaOiabdIha4naaBaaaleaacqWGPbqAcqWGQbGAaeqaaOGaeiiFaWNaeGimaaJaeiilaWIae83Wdm3aa0baaSqaaiabicdaWiabdMgaPbqaaiabikdaYaaakiabcMcaPaWcbaGaemOAaOgabeqdcqGHpis1aOGaeiilaWIaaCzcaiaaxMaadaqadiqaaiabikdaYaGaayjkaiaawMcaaaaa@52E1@

H1:g(Xi;θi)=∏jN(xij|μi(yj),σ1i2),     (3)
 MathType@MTEF@5@5@+=feaafiart1ev1aaatCvAUfKttLearuWrP9MDH5MBPbIqV92AaeXatLxBI9gBaebbnrfifHhDYfgasaacH8akY=wiFfYdH8Gipec8Eeeu0xXdbba9frFj0=OqFfea0dXdd9vqai=hGuQ8kuc9pgc9s8qqaq=dirpe0xb9q8qiLsFr0=vr0=vr0dc8meaabaqaciaacaGaaeqabaqabeGadaaakeaacqWGibasdaWgaaWcbaGaeGymaedabeaakiabcQda6iabdEgaNjabcIcaOiabdIfaynaaBaaaleaacqWGPbqAaeqaaOGaei4oaSdcciGae8hUde3aaSbaaSqaaiabdMgaPbqabaGccqGGPaqkcqGH9aqpdaqeqbqaaiabd6eaojabcIcaOiabdIha4naaBaaaleaacqWGPbqAcqWGQbGAaeqaaOGaeiiFaWNae8hVd02aaSbaaSqaaiabdMgaPbqabaGccqGGOaakcqWG5bqEdaWgaaWcbaGaemOAaOgabeaakiabcMcaPiabcYcaSiab=n8aZnaaDaaaleaacqaIXaqmcqWGPbqAaeaacqaIYaGmaaGccqGGPaqkaSqaaiabdQgaQbqab0Gaey4dIunakiabcYcaSiaaxMaacaWLjaWaaeWaceaacqaIZaWmaiaawIcacaGLPaaaaaa@59FA@

where *φ*_*i *_= {σ0i2
 MathType@MTEF@5@5@+=feaafiart1ev1aaatCvAUfKttLearuWrP9MDH5MBPbIqV92AaeXatLxBI9gBaebbnrfifHhDYfgasaacH8akY=wiFfYdH8Gipec8Eeeu0xXdbba9frFj0=OqFfea0dXdd9vqai=hGuQ8kuc9pgc9s8qqaq=dirpe0xb9q8qiLsFr0=vr0=vr0dc8meaabaqaciaacaGaaeqabaqabeGadaaakeaaiiGacqWFdpWCdaqhaaWcbaGaeGimaaJaemyAaKgabaGaeGOmaidaaaaa@31DE@} and *θ*_*i *_= {*μ*_*i*_(1), *μ*_*i*_(2),σ1i2
 MathType@MTEF@5@5@+=feaafiart1ev1aaatCvAUfKttLearuWrP9MDH5MBPbIqV92AaeXatLxBI9gBaebbnrfifHhDYfgasaacH8akY=wiFfYdH8Gipec8Eeeu0xXdbba9frFj0=OqFfea0dXdd9vqai=hGuQ8kuc9pgc9s8qqaq=dirpe0xb9q8qiLsFr0=vr0=vr0dc8meaabaqaciaacaGaaeqabaqabeGadaaakeaaiiGacqWFdpWCdaqhaaWcbaGaeGymaeJaemyAaKgabaGaeGOmaidaaaaa@31E0@} are the parameters of the null and alternative models, respectively. *N*(*x*|*μ*, *σ*^2^) denotes a normal density function with a mean *μ *and a variance *σ*^2^. *μ*_*i*_(1) and *μ*_*i*_(2) are the centers of normal distributions for *y*_*j *_= 1 (class 1) and *y*_*j *_= 2 (class 2), respectively. σ0i2
 MathType@MTEF@5@5@+=feaafiart1ev1aaatCvAUfKttLearuWrP9MDH5MBPbIqV92AaeXatLxBI9gBaebbnrfifHhDYfgasaacH8akY=wiFfYdH8Gipec8Eeeu0xXdbba9frFj0=OqFfea0dXdd9vqai=hGuQ8kuc9pgc9s8qqaq=dirpe0xb9q8qiLsFr0=vr0=vr0dc8meaabaqaciaacaGaaeqabaqabeGadaaakeaaiiGacqWFdpWCdaqhaaWcbaGaeGimaaJaemyAaKgabaGaeGOmaidaaaaa@31DE@ and σ1i2
 MathType@MTEF@5@5@+=feaafiart1ev1aaatCvAUfKttLearuWrP9MDH5MBPbIqV92AaeXatLxBI9gBaebbnrfifHhDYfgasaacH8akY=wiFfYdH8Gipec8Eeeu0xXdbba9frFj0=OqFfea0dXdd9vqai=hGuQ8kuc9pgc9s8qqaq=dirpe0xb9q8qiLsFr0=vr0=vr0dc8meaabaqaciaacaGaaeqabaqabeGadaaakeaaiiGacqWFdpWCdaqhaaWcbaGaeGymaeJaemyAaKgabaGaeGOmaidaaaaa@31E0@ are intra-class variances under the null and alternative hypotheses, respectively. For simplicity in our discussion, we assume that the expression data are normalized such that (1/*M*) ∑j=1Mxij=1
 MathType@MTEF@5@5@+=feaafiart1ev1aaatCvAUfKttLearuWrP9MDH5MBPbIqV92AaeXatLxBI9gBaebbnrfifHhDYfgasaacH8akY=wiFfYdH8Gipec8Eeeu0xXdbba9frFj0=OqFfea0dXdd9vqai=hGuQ8kuc9pgc9s8qqaq=dirpe0xb9q8qiLsFr0=vr0=vr0dc8meaabaqaciaacaGaaeqabaqabeGadaaakeaadaaeWaqaaiabdIha4naaBaaaleaacqWGPbqAcqWGQbGAaeqaaOGaeyypa0JaeGymaedaleaacqWGQbGAcqGH9aqpcqaIXaqmaeaacqWGnbqta0GaeyyeIuoaaaa@3981@ holds. In this case, the log likelihood ratio can define a significance score of a single gene *i*:

LR-S(Xi)=∑j=1Mln⁡N(xij|μi(yj),σ1i2)N(xij|0,σ0i2),     (4)
 MathType@MTEF@5@5@+=feaafiart1ev1aaatCvAUfKttLearuWrP9MDH5MBPbIqV92AaeXatLxBI9gBaebbnrfifHhDYfgasaacH8akY=wiFfYdH8Gipec8Eeeu0xXdbba9frFj0=OqFfea0dXdd9vqai=hGuQ8kuc9pgc9s8qqaq=dirpe0xb9q8qiLsFr0=vr0=vr0dc8meaabaqaciaacaGaaeqabaqabeGadaaakeaacqqGmbatcqqGsbGucqqGTaqlcqqGtbWucqGGOaakcqWGybawdaWgaaWcbaGaemyAaKgabeaakiabcMcaPiabg2da9maaqahabaGagiiBaWMaeiOBa42aaSaaaeaacqWGobGtcqGGOaakcqWG4baEdaWgaaWcbaGaemyAaKMaemOAaOgabeaakiabcYha8HGaciab=X7aTnaaBaaaleaacqWGPbqAaeqaaOGaeiikaGIaemyEaK3aaSbaaSqaaiabdQgaQbqabaGccqGGPaqkcqGGSaalcqWFdpWCdaqhaaWcbaGaeGymaeJaemyAaKgabaGaeGOmaidaaOGaeiykaKcabaGaemOta4KaeiikaGIaemiEaG3aaSbaaSqaaiabdMgaPjabdQgaQbqabaGccqGG8baFcqaIWaamcqGGSaalcqWFdpWCdaqhaaWcbaGaeGimaaJaemyAaKgabaGaeGOmaidaaOGaeiykaKcaaaWcbaGaemOAaOMaeyypa0JaeGymaedabaGaemyta0eaniabggHiLdGccqGGSaalcaWLjaGaaCzcamaabmGabaGaeGinaqdacaGLOaGaayzkaaaaaa@6B5D@

where the maximum likelihood estimates (MLEs) of the parameters are given as: μ^i(k)=∑j=1MxijI(yj=k)∑j=1MI(yj=k)
 MathType@MTEF@5@5@+=feaafiart1ev1aaatCvAUfKttLearuWrP9MDH5MBPbIqV92AaeXatLxBI9gBaebbnrfifHhDYfgasaacH8akY=wiFfYdH8Gipec8Eeeu0xXdbba9frFj0=OqFfea0dXdd9vqai=hGuQ8kuc9pgc9s8qqaq=dirpe0xb9q8qiLsFr0=vr0=vr0dc8meaabaqaciaacaGaaeqabaqabeGadaaakeaaiiGacuWF8oqBgaqcamaaBaaaleaacqWGPbqAaeqaaOGaeiikaGIaem4AaSMaeiykaKIaeyypa0ZaaSaaaeaadaaeWaqaaiabdIha4naaBaaaleaacqWGPbqAcqWGQbGAaeqaaOGaemysaKKaeiikaGIaemyEaK3aaSbaaSqaaiabdQgaQbqabaGccqGH9aqpcqWGRbWAcqGGPaqkaSqaaiabdQgaQjabg2da9iabigdaXaqaaiabd2eanbqdcqGHris5aaGcbaWaaabmaeaacqWGjbqscqGGOaakcqWG5bqEdaWgaaWcbaGaemOAaOgabeaakiabg2da9iabdUgaRjabcMcaPaWcbaGaemOAaOMaeyypa0JaeGymaedabaGaemyta0eaniabggHiLdaaaaaa@5612@, (*k *= 1, 2), σ^0i2=1M∑j=1Mxij2
 MathType@MTEF@5@5@+=feaafiart1ev1aaatCvAUfKttLearuWrP9MDH5MBPbIqV92AaeXatLxBI9gBaebbnrfifHhDYfgasaacH8akY=wiFfYdH8Gipec8Eeeu0xXdbba9frFj0=OqFfea0dXdd9vqai=hGuQ8kuc9pgc9s8qqaq=dirpe0xb9q8qiLsFr0=vr0=vr0dc8meaabaqaciaacaGaaeqabaqabeGadaaakeaaiiGacuWFdpWCgaqcamaaDaaaleaacqaIWaamcqWGPbqAaeaacqaIYaGmaaGccqGH9aqpdaWcaaqaaiabigdaXaqaaiabd2eanbaadaaeWaqaaiabdIha4naaDaaaleaacqWGPbqAcqWGQbGAaeaacqaIYaGmaaaabaGaemOAaOMaeyypa0JaeGymaedabaGaemyta0eaniabggHiLdaaaa@40DE@, and σ^1i2=1M∑j=1M(xij−μ^i(yj))2
 MathType@MTEF@5@5@+=feaafiart1ev1aaatCvAUfKttLearuWrP9MDH5MBPbIqV92AaeXatLxBI9gBaebbnrfifHhDYfgasaacH8akY=wiFfYdH8Gipec8Eeeu0xXdbba9frFj0=OqFfea0dXdd9vqai=hGuQ8kuc9pgc9s8qqaq=dirpe0xb9q8qiLsFr0=vr0=vr0dc8meaabaqaciaacaGaaeqabaqabeGadaaakeaaiiGacuWFdpWCgaqcamaaDaaaleaacqaIXaqmcqWGPbqAaeaacqaIYaGmaaGccqGH9aqpdaWcaaqaaiabigdaXaqaaiabd2eanbaadaaeWaqaaiabcIcaOiabdIha4naaBaaaleaacqWGPbqAcqWGQbGAaeqaaOGaeyOeI0Iaf8hVd0MbaKaadaWgaaWcbaGaemyAaKgabeaakiabcIcaOiabdMha5naaBaaaleaacqWGQbGAaeqaaOGaeiykaKIaeiykaKYaaWbaaSqabeaacqaIYaGmaaaabaGaemOAaOMaeyypa0JaeGymaedabaGaemyta0eaniabggHiLdaaaa@4BC7@. Here, *I*(*A*) is an index function that outputs 1 when condition *A *is satisfied; otherwise, the output is 0. By assigning the MLE to the significance score, the following estimated log likelihood ratio function is available as a score:

LR-S(Xi)=12ln⁡(σ^0i2/σ^1i2).     (5)
 MathType@MTEF@5@5@+=feaafiart1ev1aaatCvAUfKttLearuWrP9MDH5MBPbIqV92AaeXatLxBI9gBaebbnrfifHhDYfgasaacH8akY=wiFfYdH8Gipec8Eeeu0xXdbba9frFj0=OqFfea0dXdd9vqai=hGuQ8kuc9pgc9s8qqaq=dirpe0xb9q8qiLsFr0=vr0=vr0dc8meaabaqaciaacaGaaeqabaqabeGadaaakeaacqqGmbatcqqGsbGucqqGTaqlcqqGtbWucqGGOaakcqWGybawdaWgaaWcbaGaemyAaKgabeaakiabcMcaPiabg2da9maalaaabaGaeGymaedabaGaeGOmaidaaiGbcYgaSjabc6gaUnaabmGabaacciGaf83WdmNbaKaadaqhaaWcbaGaeGimaaJaemyAaKgabaGaeGOmaidaaOGaei4la8Iaf83WdmNbaKaadaqhaaWcbaGaeGymaeJaemyAaKgabaGaeGOmaidaaaGccaGLOaGaayzkaaGaeiOla4IaaCzcaiaaxMaadaqadiqaaiabiwda1aGaayjkaiaawMcaaaaa@4CE8@

Accordingly, the supervised score LR-S(*X*_*i*_) is specified by the signal-to-noise (S/N) ratio σ^0i2/σ^1i2
 MathType@MTEF@5@5@+=feaafiart1ev1aaatCvAUfKttLearuWrP9MDH5MBPbIqV92AaeXatLxBI9gBaebbnrfifHhDYfgasaacH8akY=wiFfYdH8Gipec8Eeeu0xXdbba9frFj0=OqFfea0dXdd9vqai=hGuQ8kuc9pgc9s8qqaq=dirpe0xb9q8qiLsFr0=vr0=vr0dc8meaabaqaciaacaGaaeqabaqabeGadaaakeaaiiGacuWFdpWCgaqcamaaDaaaleaacqaIWaamcqWGPbqAaeaacqaIYaGmaaGccqGGVaWlcuWFdpWCgaqcamaaDaaaleaacqaIXaqmcqWGPbqAaeaacqaIYaGmaaaaaa@3816@. The estimated likelihood ratio score for parametric hypothetical models is not necessarily the most powerful, as stated in the Neyman-Pearson's lemma, because of the variance in the parameter estimation. Although it becomes the most powerful asymptotically as the number of samples grows to infinity, there may be a better score if the number of samples is finite.

## Models and algorithms

### Unsupervised and semi-supervised likelihood models

For an active gene, gene expression is assumed to relate to the condition of each sample; in other words, it is "on" in samples in certain conditions and "off" in others. Let a hidden variable

*Z*_*i *_= (*z*_*i*1_, ..., *z*_*iM*_), *z*_*ij *_∈ {1, 2}, *j *= 1, ..., *M *denote whether the gene *i *is "on," *z*_*ij *_= 1, or "off," *z*_*ij *_= 2, in a sample *j*. In particular, we consider the binary categorization of conditions but allow uncertainty. Namely, the supervised label *y *is either 1 (class 1), 2 (class 2), or 0 (label unknown). An unsupervised case is defined as *y *= 0 for all *j*, and a semi-supervised case is defined as *y *= 0 for only some *j*.

#### Unsupervised significance score

For an active gene *i*, we assume a mixture of normal distributions for its expression *x*_*ij *_in a sample *j*:

p(xij|θi)=∑k=12P(zij=k|θi)p(xij|zij=k,θi)=∑k=12ν(k)N(xij+μ(k),σ12),     (6)
 MathType@MTEF@5@5@+=feaafiart1ev1aaatCvAUfKttLearuWrP9MDH5MBPbIqV92AaeXatLxBI9gBaebbnrfifHhDYfgasaacH8akY=wiFfYdH8Gipec8Eeeu0xXdbba9frFj0=OqFfea0dXdd9vqai=hGuQ8kuc9pgc9s8qqaq=dirpe0xb9q8qiLsFr0=vr0=vr0dc8meaabaqaciaacaGaaeqabaqabeGadaaakeaafaqabeGabaaabaGaemiCaaNaeiikaGIaemiEaG3aaSbaaSqaaiabdMgaPjabdQgaQbqabaGccqGG8baFiiGacqWF4oqCdaWgaaWcbaGaemyAaKgabeaakiabcMcaPiabg2da9maaqahabaGaemiuaaLaeiikaGIaemOEaO3aaSbaaSqaaiabdMgaPjabdQgaQbqabaGccqGH9aqpcqWGRbWAcqGG8baFcqWF4oqCdaWgaaWcbaGaemyAaKgabeaakiabcMcaPiabdchaWjabcIcaOiabdIha4naaBaaaleaacqWGPbqAcqWGQbGAaeqaaOGaeiiFaWNaemOEaO3aaSbaaSqaaiabdMgaPjabdQgaQbqabaGccqGH9aqpcqWGRbWAcqGGSaalcqWF4oqCdaWgaaWcbaGaemyAaKgabeaakiabcMcaPaWcbaGaem4AaSMaeyypa0JaeGymaedabaGaeGOmaidaniabggHiLdaakeaacqGH9aqpdaaeWbqaaiab=17aUjabcIcaOiabdUgaRjabcMcaPiabd6eaojabcIcaOiabdIha4naaBaaaleaacqWGPbqAcqWGQbGAaeqaaOGaey4kaSIae8hVd0MaeiikaGIaem4AaSMaeiykaKIaeiilaWIae83Wdm3aa0baaSqaaiabigdaXaqaaiabikdaYaaakiabcMcaPaWcbaGaem4AaSMaeyypa0JaeGymaedabaGaeGOmaidaniabggHiLdGccqGGSaalaaGaaCzcaiaaxMaadaqadiqaaiabiAda2aGaayjkaiaawMcaaaaa@85A2@

where *ν*(*k*) is the prior probability that the hidden variable *z*_*ij *_takes *k *∈ {1, 2}. We assume *ν*(*k*) is independent of *i *or *j*. Under the alternative hypothesis *H*_1_, the likelihood function of the parameter *θ*_*i *_= (*ν*(1), *ν*(2), *μ*(1), *μ*(2), σ12
 MathType@MTEF@5@5@+=feaafiart1ev1aaatCvAUfKttLearuWrP9MDH5MBPbIqV92AaeXatLxBI9gBaebbnrfifHhDYfgasaacH8akY=wiFfYdH8Gipec8Eeeu0xXdbba9frFj0=OqFfea0dXdd9vqai=hGuQ8kuc9pgc9s8qqaq=dirpe0xb9q8qiLsFr0=vr0=vr0dc8meaabaqaciaacaGaaeqabaqabeGadaaakeaaiiGacqWFdpWCdaqhaaWcbaGaeGymaedabaGaeGOmaidaaaaa@3085@), given the expression vector *X*_*i *_≡ {*x*_*i*1_,..., *x*_*iM*_} of gene *i*, is given by

g(Xi;θi)=∏j=1M∑k=12ν(k)N(xij|μ(k),σ12).     (7)
 MathType@MTEF@5@5@+=feaafiart1ev1aaatCvAUfKttLearuWrP9MDH5MBPbIqV92AaeXatLxBI9gBaebbnrfifHhDYfgasaacH8akY=wiFfYdH8Gipec8Eeeu0xXdbba9frFj0=OqFfea0dXdd9vqai=hGuQ8kuc9pgc9s8qqaq=dirpe0xb9q8qiLsFr0=vr0=vr0dc8meaabaqaciaacaGaaeqabaqabeGadaaakeaacqWGNbWzcqGGOaakcqWGybawdaWgaaWcbaGaemyAaKgabeaakiabcUda7GGaciab=H7aXnaaBaaaleaacqWGPbqAaeqaaOGaeiykaKIaeyypa0ZaaebCaeaadaaeWbqaaiab=17aUjabcIcaOiabdUgaRjabcMcaPiabd6eaojabcIcaOiabdIha4naaBaaaleaacqWGPbqAcqWGQbGAaeqaaOGaeiiFaWNae8hVd0MaeiikaGIaem4AaSMaeiykaKIaeiilaWIae83Wdm3aa0baaSqaaiabigdaXaqaaiabikdaYaaakiabcMcaPaWcbaGaem4AaSMaeyypa0JaeGymaedabaGaeGOmaidaniabggHiLdaaleaacqWGQbGAcqGH9aqpcqaIXaqmaeaacqWGnbqta0Gaey4dIunakiabc6caUiaaxMaacaWLjaWaaeWaceaacqaI3aWnaiaawIcacaGLPaaaaaa@60B5@

The null hypothesis is the same as given in the supervised case, i.e., a normal distribution:

H0:f(Xi;φi)=∏jN(xij|0,σ02).     (8)
 MathType@MTEF@5@5@+=feaafiart1ev1aaatCvAUfKttLearuWrP9MDH5MBPbIqV92AaeXatLxBI9gBaebbnrfifHhDYfgasaacH8akY=wiFfYdH8Gipec8Eeeu0xXdbba9frFj0=OqFfea0dXdd9vqai=hGuQ8kuc9pgc9s8qqaq=dirpe0xb9q8qiLsFr0=vr0=vr0dc8meaabaqaciaacaGaaeqabaqabeGadaaakeaacqWGibasdaWgaaWcbaGaeGimaadabeaakiabcQda6iabdAgaMjabcIcaOiabdIfaynaaBaaaleaacqWGPbqAaeqaaOGaei4oaSdcciGae8NXdy2aaSbaaSqaaiabdMgaPbqabaGccqGGPaqkcqGH9aqpdaqeqbqaaiabd6eaobWcbaGaemOAaOgabeqdcqGHpis1aOGaeiikaGIaemiEaG3aaSbaaSqaaiabdMgaPjabdQgaQbqabaGccqGG8baFcqaIWaamcqGGSaalcqWFdpWCdaqhaaWcbaGaeGimaadabaGaeGOmaidaaOGaeiykaKIaeiOla4IaaCzcaiaaxMaadaqadiqaaiabiIda4aGaayjkaiaawMcaaaaa@5196@

The log-likelihood ratio score is then defined as

LR-U(Xi)=ln⁡g(Xi;φ^i)f(Xi;θ^i),     (9)
 MathType@MTEF@5@5@+=feaafiart1ev1aaatCvAUfKttLearuWrP9MDH5MBPbIqV92AaeXatLxBI9gBaebbnrfifHhDYfgasaacH8akY=wiFfYdH8Gipec8Eeeu0xXdbba9frFj0=OqFfea0dXdd9vqai=hGuQ8kuc9pgc9s8qqaq=dirpe0xb9q8qiLsFr0=vr0=vr0dc8meaabaqaciaacaGaaeqabaqabeGadaaakeaacqqGmbatcqqGsbGucqqGTaqlcqqGvbqvcqGGOaakcqWGybawdaWgaaWcbaGaemyAaKgabeaakiabcMcaPiabg2da9iGbcYgaSjabc6gaUnaalaaabaGaem4zaCMaeiikaGIaemiwaG1aaSbaaSqaaiabdMgaPbqabaGccqGG7aWoiiGacuWFgpGzgaqcamaaBaaaleaacqWGPbqAaeqaaOGaeiykaKcabaGaemOzayMaeiikaGIaemiwaG1aaSbaaSqaaiabdMgaPbqabaGccqGG7aWocuWF4oqCgaqcamaaBaaaleaacqWGPbqAaeqaaOGaeiykaKcaaiabcYcaSiaaxMaacaWLjaWaaeWaceaacqaI5aqoaiaawIcacaGLPaaaaaa@5262@

where φ^i=arg⁡max⁡φi
 MathType@MTEF@5@5@+=feaafiart1ev1aaatCvAUfKttLearuWrP9MDH5MBPbIqV92AaeXatLxBI9gBaebbnrfifHhDYfgasaacH8akY=wiFfYdH8Gipec8Eeeu0xXdbba9frFj0=OqFfea0dXdd9vqai=hGuQ8kuc9pgc9s8qqaq=dirpe0xb9q8qiLsFr0=vr0=vr0dc8meaabaqaciaacaGaaeqabaqabeGadaaakeaaiiGacuWFgpGzgaqcamaaBaaaleaacqWGPbqAaeqaaOGaeyypa0JagiyyaeMaeiOCaiNaei4zaCMagiyBa0MaeiyyaeMaeiiEaG3aaSbaaSqaaiab=z8aMnaaBaaameaacqWGPbqAaeqaaaWcbeaaaaa@3CBA@*f *(*X*_*i*_; *φ*_*i*_) and θ^i=arg⁡max⁡θi
 MathType@MTEF@5@5@+=feaafiart1ev1aaatCvAUfKttLearuWrP9MDH5MBPbIqV92AaeXatLxBI9gBaebbnrfifHhDYfgasaacH8akY=wiFfYdH8Gipec8Eeeu0xXdbba9frFj0=OqFfea0dXdd9vqai=hGuQ8kuc9pgc9s8qqaq=dirpe0xb9q8qiLsFr0=vr0=vr0dc8meaabaqaciaacaGaaeqabaqabeGadaaakeaaiiGacuWF4oqCgaqcamaaBaaaleaacqWGPbqAaeqaaOGaeyypa0JagiyyaeMaeiOCaiNaei4zaCMagiyBa0MaeiyyaeMaeiiEaG3aaSbaaSqaaiab=H7aXnaaBaaameaacqWGPbqAaeqaaaWcbeaaaaa@3CB4@*g*(*X*_*i*_; *θ*_*i*_) are the MLEs for the two hypotheses.

When we have an MLE θ^
 MathType@MTEF@5@5@+=feaafiart1ev1aaatCvAUfKttLearuWrP9MDH5MBPbIqV92AaeXatLxBI9gBaebbnrfifHhDYfgasaacH8akY=wiFfYdH8Gipec8Eeeu0xXdbba9frFj0=OqFfea0dXdd9vqai=hGuQ8kuc9pgc9s8qqaq=dirpe0xb9q8qiLsFr0=vr0=vr0dc8meaabaqaciaacaGaaeqabaqabeGadaaakeaaiiGacuWF4oqCgaqcaaaa@2E79@ for the alternative hypothesis, the posterior of the hidden variable *z*_*ij*_, given a datum *x*_*ij*_, is obtained as

z^ij(k)=defP(zij=k|xij,θ^i)=ν(k)N(xij|μ(k),σ12)∑k=12ν(k)N(xij|μ(k),σ12).     (10)
 MathType@MTEF@5@5@+=feaafiart1ev1aaatCvAUfKttLearuWrP9MDH5MBPbIqV92AaeXatLxBI9gBaebbnrfifHhDYfgasaacH8akY=wiFfYdH8Gipec8Eeeu0xXdbba9frFj0=OqFfea0dXdd9vqai=hGuQ8kuc9pgc9s8qqaq=dirpe0xb9q8qiLsFr0=vr0=vr0dc8meaabaqaciaacaGaaeqabaqabeGadaaakeaacuWG6bGEgaqcamaaBaaaleaacqWGPbqAcqWGQbGAaeqaaOGaeiikaGIaem4AaSMaeiykaKYaaCbiaeaacqGH9aqpaSqabeaacqqGKbazcqqGLbqzcqqGMbGzaaGccqWGqbaucqGGOaakcqWG6bGEdaWgaaWcbaGaemyAaKMaemOAaOgabeaakiabg2da9iabdUgaRjabcYha8jabdIha4naaBaaaleaacqWGPbqAcqWGQbGAaeqaaOGaeiilaWccciGaf8hUdeNbaKaadaWgaaWcbaGaemyAaKgabeaakiabcMcaPiabg2da9maalaaabaGae8xVd4MaeiikaGIaem4AaSMaeiykaKIaemOta4KaeiikaGIaemiEaG3aaSbaaSqaaiabdMgaPjabdQgaQbqabaGccqGG8baFcqWF8oqBcqGGOaakcqWGRbWAcqGGPaqkcqGGSaalcqWFdpWCdaqhaaWcbaGaeGymaedabaGaeGOmaidaaOGaeiykaKcabaWaaabmaeaacqWF9oGBcqGGOaakcqWGRbWAcqGGPaqkcqWGobGtcqGGOaakcqWG4baEdaWgaaWcbaGaemyAaKMaemOAaOgabeaakiabcYha8jab=X7aTjabcIcaOiabdUgaRjabcMcaPiabcYcaSiab=n8aZnaaDaaaleaacqaIXaqmaeaacqaIYaGmaaGccqGGPaqkaSqaaiabdUgaRjabg2da9iabigdaXaqaaiabikdaYaqdcqGHris5aaaakiabc6caUiaaxMaacaWLjaWaaeWaceaacqaIXaqmcqaIWaamaiaawIcacaGLPaaaaaa@8844@

Let Z^i
 MathType@MTEF@5@5@+=feaafiart1ev1aaatCvAUfKttLearuWrP9MDH5MBPbIqV92AaeXatLxBI9gBaebbnrfifHhDYfgasaacH8akY=wiFfYdH8Gipec8Eeeu0xXdbba9frFj0=OqFfea0dXdd9vqai=hGuQ8kuc9pgc9s8qqaq=dirpe0xb9q8qiLsFr0=vr0=vr0dc8meaabaqaciaacaGaaeqabaqabeGadaaakeaacuWGAbGwgaqcamaaBaaaleaacqWGPbqAaeqaaaaa@2F80@ = (z^ij
 MathType@MTEF@5@5@+=feaafiart1ev1aaatCvAUfKttLearuWrP9MDH5MBPbIqV92AaeXatLxBI9gBaebbnrfifHhDYfgasaacH8akY=wiFfYdH8Gipec8Eeeu0xXdbba9frFj0=OqFfea0dXdd9vqai=hGuQ8kuc9pgc9s8qqaq=dirpe0xb9q8qiLsFr0=vr0=vr0dc8meaabaqaciaacaGaaeqabaqabeGadaaakeaacuWG6bGEgaqcamaaBaaaleaacqWGPbqAcqWGQbGAaeqaaaaa@311D@ (*k*); *j *= 1, ..., *M*, *k *= 1, 2) be the matrix of the estimated posterior, where 0 ≤ z^ij
 MathType@MTEF@5@5@+=feaafiart1ev1aaatCvAUfKttLearuWrP9MDH5MBPbIqV92AaeXatLxBI9gBaebbnrfifHhDYfgasaacH8akY=wiFfYdH8Gipec8Eeeu0xXdbba9frFj0=OqFfea0dXdd9vqai=hGuQ8kuc9pgc9s8qqaq=dirpe0xb9q8qiLsFr0=vr0=vr0dc8meaabaqaciaacaGaaeqabaqabeGadaaakeaacuWG6bGEgaqcamaaBaaaleaacqWGPbqAcqWGQbGAaeqaaaaa@311D@ (*k*) ≤ 1 and ∑_*k *_z^ij
 MathType@MTEF@5@5@+=feaafiart1ev1aaatCvAUfKttLearuWrP9MDH5MBPbIqV92AaeXatLxBI9gBaebbnrfifHhDYfgasaacH8akY=wiFfYdH8Gipec8Eeeu0xXdbba9frFj0=OqFfea0dXdd9vqai=hGuQ8kuc9pgc9s8qqaq=dirpe0xb9q8qiLsFr0=vr0=vr0dc8meaabaqaciaacaGaaeqabaqabeGadaaakeaacuWG6bGEgaqcamaaBaaaleaacqWGPbqAcqWGQbGAaeqaaaaa@311D@(*k*) = 1 hold. The matrix Z^i
 MathType@MTEF@5@5@+=feaafiart1ev1aaatCvAUfKttLearuWrP9MDH5MBPbIqV92AaeXatLxBI9gBaebbnrfifHhDYfgasaacH8akY=wiFfYdH8Gipec8Eeeu0xXdbba9frFj0=OqFfea0dXdd9vqai=hGuQ8kuc9pgc9s8qqaq=dirpe0xb9q8qiLsFr0=vr0=vr0dc8meaabaqaciaacaGaaeqabaqabeGadaaakeaacuWGAbGwgaqcamaaBaaaleaacqWGPbqAaeqaaaaa@2F80@ will be used later when we consider multiple testing.

#### Semi-supervised significance score

In the semi-supervised case, an active gene can be modeled by modifying the prior of the hidden variable so that the prior incorporates the label's inobservability:

*P*(*z*_*ij *_= *k*|*y*_*j*_, *θ*_*i*_) = *I*(*y*_*j *_= *k*) + *I*(*y*_*j *_= 0)*ν*(*k*), *k *= 1, 2.     (11)

This prior states that the hidden label is the same as the observed label when it is observed, but is predominantly predicted by the prior knowledge *ν*(*k*) when it is unobserved; the point is, we regard the observation as prior information. Accordingly, a semi-supervised case is dealt with by simply employing the same prior as in the unsupervised case, Eq. (11), but the first term vanishes because *I*(*y*_*j *_= 0) for every *j*. Namely, the log-likelihood ratio score in a semi-supervised case, LR-SS(*X*_*i*_), is defined as in the unsupervised case except that the prior *P*(*z*_*ij *_= *k*|*θ*_*i*_)in Eq. (6) is replaced by *P*(*z*_*ij *_= *k*|*y*_*j*_, *θ*_*i*_) in Eq. (11).

### Optimal discovery procedure

#### ODP lemma and its application

According to Neyman-Pearson's lemma, a statistic is defined as being most powerful when its detection probability *β *is the largest with a fixed significance rate *α*, and the likelihood ratio is proven to be the most powerful when the null and alternative hypotheses are both simple. Storey et al. (2005) [6,7] extended Neyman-Pearson's framework to multiple testing and proposed a new general framework, ODP. They defined that a statistic is optimal if it maximizes the expected true positive (ETP) rate when fixing the expected false positive (EFP) rate. They also showed that an ODP function is available as in the following ODP lemma, which is not the same as the likelihood ratio.

#### ODP lemma

*Let S*_ODP _*be a common statistic, called an ODP function, for all genes:*

SODP(X)=∑i′∈G1g(X|θi′)∑i′∈G0f(X|φi′),     (12)
 MathType@MTEF@5@5@+=feaafiart1ev1aaatCvAUfKttLearuWrP9MDH5MBPbIqV92AaeXatLxBI9gBaebbnrfifHhDYfgasaacH8akY=wiFfYdH8Gipec8Eeeu0xXdbba9frFj0=OqFfea0dXdd9vqai=hGuQ8kuc9pgc9s8qqaq=dirpe0xb9q8qiLsFr0=vr0=vr0dc8meaabaqaciaacaGaaeqabaqabeGadaaakeaacqWGtbWudaWgaaWcbaGaee4ta8KaeeiraqKaeeiuaafabeaakiabcIcaOiabdIfayjabcMcaPiabg2da9maalaaabaWaaabeaeaacqWGNbWzcqGGOaakcqWGybawcqGG8baFiiGacqWF4oqCdaWgaaWcbaGafmyAaKMbauaaaeqaaOGaeiykaKcaleaacuWGPbqAgaqbaiabgIGiolabdEeahnaaBaaameaacqaIXaqmaeqaaaWcbeqdcqGHris5aaGcbaWaaabeaeaacqWGMbGzcqGGOaakcqWGybawcqGG8baFcqWFgpGzdaWgaaWcbaGafmyAaKMbauaaaeqaaOGaeiykaKcaleaacuWGPbqAgaqbaiabgIGiolabdEeahnaaBaaameaacqaIWaamaeqaaaWcbeqdcqGHris5aaaakiabcYcaSiaaxMaacaWLjaWaaeWaceaacqaIXaqmcqaIYaGmaiaawIcacaGLPaaaaaa@5B57@

*where X is a gene expression vector *(*its dimensionality corresponds to the number of samples*). *Then, the criterion that the gene is significant when S*_ODP _(*X*_*i*_) > *λ for any threshold λ *> 0 *is an ODP. In Eq*. (*12*)*, G*_0 _*and G*_1 _*are index sets of inactive and active genes, respectively*.

This lemma defines an ideal ODP but not a practical one, because it needs information not available in actual situations of multiple testing. The true values of parameters *θ *and *φ *are not available and are instead substituted by MLEs estimated from the observed data. Furthermore, *G*_0 _and *G*_1 _are not available in a real situation either, because there is no need to calculate significance scores if we know *G*_0 _and *G*_1_. For practical use, therefore, Storey et al. proposed an approximate ODP criterion:

S^ODP(Xi)=∑i′g(Xi|θ^i′)∑i′∈G^0f(Xi|φ^i′),     (13)
 MathType@MTEF@5@5@+=feaafiart1ev1aaatCvAUfKttLearuWrP9MDH5MBPbIqV92AaeXatLxBI9gBaebbnrfifHhDYfgasaacH8akY=wiFfYdH8Gipec8Eeeu0xXdbba9frFj0=OqFfea0dXdd9vqai=hGuQ8kuc9pgc9s8qqaq=dirpe0xb9q8qiLsFr0=vr0=vr0dc8meaabaqaciaacaGaaeqabaqabeGadaaakeaacuWGtbWugaqcamaaBaaaleaacqqGpbWtcqqGebarcqqGqbauaeqaaOGaeiikaGIaemiwaG1aaSbaaSqaaiabdMgaPbqabaGccqGGPaqkcqGH9aqpdaWcaaqaamaaqababaGaem4zaCMaeiikaGIaemiwaG1aaSbaaSqaaiabdMgaPbqabaGccqGG8baFiiGacuWF4oqCgaqcamaaBaaaleaacuWGPbqAgaqbaaqabaGccqGGPaqkaSqaaiqbdMgaPzaafaaabeqdcqGHris5aaGcbaWaaabeaeaacqWGMbGzcqGGOaakcqWGybawdaWgaaWcbaGaemyAaKgabeaakiabcYha8jqb=z8aMzaajaWaaSbaaSqaaiqbdMgaPzaafaaabeaakiabcMcaPaWcbaGafmyAaKMbauaacqGHiiIZcuWGhbWrgaqcamaaBaaameaacqaIWaamaeqaaaWcbeqdcqGHris5aaaakiabcYcaSiaaxMaacaWLjaWaaeWaceaacqaIXaqmcqaIZaWmaiaawIcacaGLPaaaaaa@5C89@

where θ^i=arg⁡max⁡θi
 MathType@MTEF@5@5@+=feaafiart1ev1aaatCvAUfKttLearuWrP9MDH5MBPbIqV92AaeXatLxBI9gBaebbnrfifHhDYfgasaacH8akY=wiFfYdH8Gipec8Eeeu0xXdbba9frFj0=OqFfea0dXdd9vqai=hGuQ8kuc9pgc9s8qqaq=dirpe0xb9q8qiLsFr0=vr0=vr0dc8meaabaqaciaacaGaaeqabaqabeGadaaakeaaiiGacuWF4oqCgaqcamaaBaaaleaacqWGPbqAaeqaaOGaeyypa0JagiyyaeMaeiOCaiNaei4zaCMagiyBa0MaeiyyaeMaeiiEaG3aaSbaaSqaaiab=H7aXnaaBaaameaacqWGPbqAaeqaaaWcbeaaaaa@3CB4@*g *(*X*_*i*_|*θ*_*i*_) and φ^i=arg⁡max⁡φi
 MathType@MTEF@5@5@+=feaafiart1ev1aaatCvAUfKttLearuWrP9MDH5MBPbIqV92AaeXatLxBI9gBaebbnrfifHhDYfgasaacH8akY=wiFfYdH8Gipec8Eeeu0xXdbba9frFj0=OqFfea0dXdd9vqai=hGuQ8kuc9pgc9s8qqaq=dirpe0xb9q8qiLsFr0=vr0=vr0dc8meaabaqaciaacaGaaeqabaqabeGadaaakeaaiiGacuWFgpGzgaqcamaaBaaaleaacqWGPbqAaeqaaOGaeyypa0JagiyyaeMaeiOCaiNaei4zaCMagiyBa0MaeiyyaeMaeiiEaG3aaSbaaSqaaiab=z8aMnaaBaaameaacqWGPbqAaeqaaaWcbeaaaaa@3CBA@*f*(*X*_*i*_|*φ*_*i*_) are the MLEs. Note here that the summation in the numerator is taken over all genes, and that the summation in the denominator is taken for a roughly estimated set of inactive genes, G^0
 MathType@MTEF@5@5@+=feaafiart1ev1aaatCvAUfKttLearuWrP9MDH5MBPbIqV92AaeXatLxBI9gBaebbnrfifHhDYfgasaacH8akY=wiFfYdH8Gipec8Eeeu0xXdbba9frFj0=OqFfea0dXdd9vqai=hGuQ8kuc9pgc9s8qqaq=dirpe0xb9q8qiLsFr0=vr0=vr0dc8meaabaqaciaacaGaaeqabaqabeGadaaakeaacuWGhbWrgaqcamaaBaaaleaacqaIWaamaeqaaaaa@2EED@. As an example, they proposed G^0
 MathType@MTEF@5@5@+=feaafiart1ev1aaatCvAUfKttLearuWrP9MDH5MBPbIqV92AaeXatLxBI9gBaebbnrfifHhDYfgasaacH8akY=wiFfYdH8Gipec8Eeeu0xXdbba9frFj0=OqFfea0dXdd9vqai=hGuQ8kuc9pgc9s8qqaq=dirpe0xb9q8qiLsFr0=vr0=vr0dc8meaabaqaciaacaGaaeqabaqabeGadaaakeaacuWGhbWrgaqcamaaBaaaleaacqaIWaamaeqaaaaa@2EED@ = {*j*|*g*(*X*_*j*_|θ^i
 MathType@MTEF@5@5@+=feaafiart1ev1aaatCvAUfKttLearuWrP9MDH5MBPbIqV92AaeXatLxBI9gBaebbnrfifHhDYfgasaacH8akY=wiFfYdH8Gipec8Eeeu0xXdbba9frFj0=OqFfea0dXdd9vqai=hGuQ8kuc9pgc9s8qqaq=dirpe0xb9q8qiLsFr0=vr0=vr0dc8meaabaqaciaacaGaaeqabaqabeGadaaakeaaiiGacuWF4oqCgaqcamaaBaaaleaacqWGPbqAaeqaaaaa@3000@)/*f*(*X*_*j*_|φ^i
 MathType@MTEF@5@5@+=feaafiart1ev1aaatCvAUfKttLearuWrP9MDH5MBPbIqV92AaeXatLxBI9gBaebbnrfifHhDYfgasaacH8akY=wiFfYdH8Gipec8Eeeu0xXdbba9frFj0=OqFfea0dXdd9vqai=hGuQ8kuc9pgc9s8qqaq=dirpe0xb9q8qiLsFr0=vr0=vr0dc8meaabaqaciaacaGaaeqabaqabeGadaaakeaaiiGacuWFgpGzgaqcamaaBaaaleaacqWGPbqAaeqaaaaa@3003@) <*ε*}, where *ε *is an arbitrary positive threshold; that is, G^0
 MathType@MTEF@5@5@+=feaafiart1ev1aaatCvAUfKttLearuWrP9MDH5MBPbIqV92AaeXatLxBI9gBaebbnrfifHhDYfgasaacH8akY=wiFfYdH8Gipec8Eeeu0xXdbba9frFj0=OqFfea0dXdd9vqai=hGuQ8kuc9pgc9s8qqaq=dirpe0xb9q8qiLsFr0=vr0=vr0dc8meaabaqaciaacaGaaeqabaqabeGadaaakeaacuWGhbWrgaqcamaaBaaaleaacqaIWaamaeqaaaaa@2EED@ is a set of genes that are not significant by the standard gene-wise likelihood ratio test.

By applying the typical ODP estimation to supervised differential gene discovery problems for artificial and real data sets, they showed that ODP is better at detecting active genes than the existing methods. This means a larger number of genes were obtained at each fixed false discovery rate (FDR), where the FDR was estimated by a permutation test.

The key of the ODP in contrast to the individual likelihood ratio is that ODP shares among all genes common information about the distributions represented as null and alternative models, so that the common information is used when evaluating a single gene. They actually demonstrated that, when the hypothetical models have some global characters shared by the genes, such as an asymmetric or cluster structure or both, ODP can incorporate them to improve the performance of gene discovery, i.e., to increase the ETP for a fixed EFP.

### ODP to latent variable models

For parametric hypothetical models, the distribution of hypotheses is attributed to the distribution of model parameters. When there are hidden variables, as in our model's case, other definitions of the hypothesis are possible: (a) unknown values of the hidden variables are marginalized out in the hypotheses, or (b) the hidden variables are explicitly included, as are the model parameters in the hypothetical models. In case (b), the distribution of hypotheses is attributed to the distributions of both model parameters and hidden variables. These two definitions of the hypothesis distribution lead to different results. We formalize these two cases in this section and compare them in the next one.

In the likelihood ratio score, we evaluate the ratio of the two likelihood functions, *g*(·|θ^i
 MathType@MTEF@5@5@+=feaafiart1ev1aaatCvAUfKttLearuWrP9MDH5MBPbIqV92AaeXatLxBI9gBaebbnrfifHhDYfgasaacH8akY=wiFfYdH8Gipec8Eeeu0xXdbba9frFj0=OqFfea0dXdd9vqai=hGuQ8kuc9pgc9s8qqaq=dirpe0xb9q8qiLsFr0=vr0=vr0dc8meaabaqaciaacaGaaeqabaqabeGadaaakeaaiiGacuWF4oqCgaqcamaaBaaaleaacqWGPbqAaeqaaaaa@3000@) and *f *(·|φ^i
 MathType@MTEF@5@5@+=feaafiart1ev1aaatCvAUfKttLearuWrP9MDH5MBPbIqV92AaeXatLxBI9gBaebbnrfifHhDYfgasaacH8akY=wiFfYdH8Gipec8Eeeu0xXdbba9frFj0=OqFfea0dXdd9vqai=hGuQ8kuc9pgc9s8qqaq=dirpe0xb9q8qiLsFr0=vr0=vr0dc8meaabaqaciaacaGaaeqabaqabeGadaaakeaaiiGacuWFgpGzgaqcamaaBaaaleaacqWGPbqAaeqaaaaa@3003@), for each gene *i'*, where we estimate the parameters of the null and alternative models, θ^i
 MathType@MTEF@5@5@+=feaafiart1ev1aaatCvAUfKttLearuWrP9MDH5MBPbIqV92AaeXatLxBI9gBaebbnrfifHhDYfgasaacH8akY=wiFfYdH8Gipec8Eeeu0xXdbba9frFj0=OqFfea0dXdd9vqai=hGuQ8kuc9pgc9s8qqaq=dirpe0xb9q8qiLsFr0=vr0=vr0dc8meaabaqaciaacaGaaeqabaqabeGadaaakeaaiiGacuWF4oqCgaqcamaaBaaaleaacqWGPbqAaeqaaaaa@3000@ and φ^i
 MathType@MTEF@5@5@+=feaafiart1ev1aaatCvAUfKttLearuWrP9MDH5MBPbIqV92AaeXatLxBI9gBaebbnrfifHhDYfgasaacH8akY=wiFfYdH8Gipec8Eeeu0xXdbba9frFj0=OqFfea0dXdd9vqai=hGuQ8kuc9pgc9s8qqaq=dirpe0xb9q8qiLsFr0=vr0=vr0dc8meaabaqaciaacaGaaeqabaqabeGadaaakeaaiiGacuWFgpGzgaqcamaaBaaaleaacqWGPbqAaeqaaaaa@3003@, respectively, as MLEs, and the hidden variable in the alternative model, as its posterior, Z^i′
 MathType@MTEF@5@5@+=feaafiart1ev1aaatCvAUfKttLearuWrP9MDH5MBPbIqV92AaeXatLxBI9gBaebbnrfifHhDYfgasaacH8akY=wiFfYdH8Gipec8Eeeu0xXdbba9frFj0=OqFfea0dXdd9vqai=hGuQ8kuc9pgc9s8qqaq=dirpe0xb9q8qiLsFr0=vr0=vr0dc8meaabaqaciaacaGaaeqabaqabeGadaaakeaacuWGAbGwgaqcamaaBaaaleaacuWGPbqAgaqbaaqabaaaaa@2F8C@. In the ODP, only a single significance function, (13), is constructed by using a set of hypotheses, each of which corresponds to a single gene; hence, the likelihood functions, *g *(·|θ^i
 MathType@MTEF@5@5@+=feaafiart1ev1aaatCvAUfKttLearuWrP9MDH5MBPbIqV92AaeXatLxBI9gBaebbnrfifHhDYfgasaacH8akY=wiFfYdH8Gipec8Eeeu0xXdbba9frFj0=OqFfea0dXdd9vqai=hGuQ8kuc9pgc9s8qqaq=dirpe0xb9q8qiLsFr0=vr0=vr0dc8meaabaqaciaacaGaaeqabaqabeGadaaakeaaiiGacuWF4oqCgaqcamaaBaaaleaacqWGPbqAaeqaaaaa@3000@) and *f *(·|φ^i
 MathType@MTEF@5@5@+=feaafiart1ev1aaatCvAUfKttLearuWrP9MDH5MBPbIqV92AaeXatLxBI9gBaebbnrfifHhDYfgasaacH8akY=wiFfYdH8Gipec8Eeeu0xXdbba9frFj0=OqFfea0dXdd9vqai=hGuQ8kuc9pgc9s8qqaq=dirpe0xb9q8qiLsFr0=vr0=vr0dc8meaabaqaciaacaGaaeqabaqabeGadaaakeaaiiGacuWFgpGzgaqcamaaBaaaleaacqWGPbqAaeqaaaaa@3003@), for gene *i' *are shared by all genes. Namely, in the ODP, we evaluate *g*(*X*_*i*_|θ^i
 MathType@MTEF@5@5@+=feaafiart1ev1aaatCvAUfKttLearuWrP9MDH5MBPbIqV92AaeXatLxBI9gBaebbnrfifHhDYfgasaacH8akY=wiFfYdH8Gipec8Eeeu0xXdbba9frFj0=OqFfea0dXdd9vqai=hGuQ8kuc9pgc9s8qqaq=dirpe0xb9q8qiLsFr0=vr0=vr0dc8meaabaqaciaacaGaaeqabaqabeGadaaakeaaiiGacuWF4oqCgaqcamaaBaaaleaacqWGPbqAaeqaaaaa@3000@) and *f*(*X*_*i*_|φ^i
 MathType@MTEF@5@5@+=feaafiart1ev1aaatCvAUfKttLearuWrP9MDH5MBPbIqV92AaeXatLxBI9gBaebbnrfifHhDYfgasaacH8akY=wiFfYdH8Gipec8Eeeu0xXdbba9frFj0=OqFfea0dXdd9vqai=hGuQ8kuc9pgc9s8qqaq=dirpe0xb9q8qiLsFr0=vr0=vr0dc8meaabaqaciaacaGaaeqabaqabeGadaaakeaaiiGacuWFgpGzgaqcamaaBaaaleaacqWGPbqAaeqaaaaa@3003@) for *i *≠ *i'*, and the key difference between cases (a) and (b) is in the way this process is carried out.

In case (a), i.e., when only the model parameters are shared by all genes, the likelihood function becomes

g^(Xi|θ^i′)=∏j=1Mp(xij|θ^i′)=∏j=1M∑k=12ν^(k)N(xij|μ^i′(k),σ^1i′2).     (14)
 MathType@MTEF@5@5@+=feaafiart1ev1aaatCvAUfKttLearuWrP9MDH5MBPbIqV92AaeXatLxBI9gBaebbnrfifHhDYfgasaacH8akY=wiFfYdH8Gipec8Eeeu0xXdbba9frFj0=OqFfea0dXdd9vqai=hGuQ8kuc9pgc9s8qqaq=dirpe0xb9q8qiLsFr0=vr0=vr0dc8meaabaqaciaacaGaaeqabaqabeGadaaakeGadmatcaaFcmaXcuaabaqaceaaaeaacuWGNbWzgaqcaiabcIcaOiabdIfaynaaBaaaleaacqWGPbqAaeqaaOGaeiiFaWhcciGaf8hUdeNbaKaadaWgaaWcbaGafmyAaKMbauaaaeqaaOGaeiykaKIaeyypa0ZaaebCaeaacqWGWbaCcqGGOaakcqWG4baEdaWgaaWcbaGaemyAaKMaemOAaOgabeaakiabcYha8jqb=H7aXzaajaWaaSbaaSqaaiqbdMgaPzaafaaabeaakiabcMcaPaWcbaGaemOAaOMaeyypa0JaeGymaedabaGaemyta0eaniabg+GivdaakeGadaqpfmawfaqKfiaaxMaacqGH9aqpdaqeWbqaamaaqahabaGaf8xVd4MbaKaacqGGOaakcqWGRbWAcqGGPaqkcqWGobGtcqGGOaakcqWG4baEdaWgaaWcbaGaemyAaKMaemOAaOgabeaakiabcYha8jqb=X7aTzaajaWaaSbaaSqaaiqbdMgaPzaafaaabeaakiabcIcaOiabdUgaRjabcMcaPiabcYcaSiqb=n8aZzaajaWaa0baaSqaaiabigdaXiqbdMgaPzaafaaabaGaeGOmaidaaOGaeiykaKcaleaacqWGRbWAcqGH9aqpcqaIXaqmaeaacqaIYaGma0GaeyyeIuoaaSqaaiabdQgaQjabg2da9iabigdaXaqaaiabd2eanbqdcqGHpis1aOGaeiOla4caaiaaxMaacaWLjaWaaeWaceaacqaIXaqmcqaI0aanaiaawIcacaGLPaaaaaa@7D20@

In case (b), i.e., when the estimated posterior of the hidden variable, Z^i′
 MathType@MTEF@5@5@+=feaafiart1ev1aaatCvAUfKttLearuWrP9MDH5MBPbIqV92AaeXatLxBI9gBaebbnrfifHhDYfgasaacH8akY=wiFfYdH8Gipec8Eeeu0xXdbba9frFj0=OqFfea0dXdd9vqai=hGuQ8kuc9pgc9s8qqaq=dirpe0xb9q8qiLsFr0=vr0=vr0dc8meaabaqaciaacaGaaeqabaqabeGadaaakeaacuWGAbGwgaqcamaaBaaaleaacuWGPbqAgaqbaaqabaaaaa@2F8C@, is also shared, the likelihood function becomes

g^(Xi|θ^i′,Z^i′)=∏j=1Mp(xij|z^i′j,θ^i′)=∏j=1M∑k=12z^i′j(k)N(xij|μ^i′(k),σ^1i′2).     (15)
 MathType@MTEF@5@5@+=feaafiart1ev1aaatCvAUfKttLearuWrP9MDH5MBPbIqV92AaeXatLxBI9gBaebbnrfifHhDYfgasaacH8akY=wiFfYdH8Gipec8Eeeu0xXdbba9frFj0=OqFfea0dXdd9vqai=hGuQ8kuc9pgc9s8qqaq=dirpe0xb9q8qiLsFr0=vr0=vr0dc8meaabaqaciaacaGaaeqabaqabeGadaaakeGadmatcaaFcmaXcuaabaqaceaaaeaacuWGNbWzgaqcaiabcIcaOiabdIfaynaaBaaaleaacqWGPbqAaeqaaOGaeiiFaWhcciGaf8hUdeNbaKaadaWgaaWcbaGafmyAaKMbauaaaeqaaOGaeiilaWIafmOwaOLbaKaadaWgaaWcbaGafmyAaKMbauaaaeqaaOGaeiykaKIaeyypa0ZaaebCaeaacqWGWbaCcqGGOaakcqWG4baEdaWgaaWcbaGaemyAaKMaemOAaOgabeaakiabcYha8jqbdQha6zaajaWaaSbaaSqaaiqbdMgaPzaafaGaemOAaOgabeaakiab=XcaSiqb=H7aXzaajaWaaSbaaSqaaiqbdMgaPzaafaaabeaakiabcMcaPaWcbaGaemOAaOMaeyypa0JaeGymaedabaGaemyta0eaniabg+GivdaakeGadaaihmaohaaMhiaaxMaacqGH9aqpdaqeWbqaamaaqahabaGafmOEaONbaKaadaWgaaWcbaGafmyAaKMbauaacqWGQbGAaeqaaOGaeiikaGIaem4AaSMaeiykaKIaemOta4KaeiikaGIaemiEaG3aaSbaaSqaaiabdMgaPjabdQgaQbqabaGccqGG8baFcuWF8oqBgaqcamaaBaaaleaacuWGPbqAgaqbaaqabaGccqGGOaakcqWGRbWAcqGGPaqkcqGGSaalcuWFdpWCgaqcamaaDaaaleaacqaIXaqmcuWGPbqAgaqbaaqaaiabikdaYaaakiabcMcaPaWcbaGaem4AaSMaeyypa0JaeGymaedabaGaeGOmaidaniabggHiLdaaleaacqWGQbGAcqGH9aqpcqaIXaqmaeaacqWGnbqta0Gaey4dIunakiabc6caUaaacaWLjaGaaCzcamaabmGabaGaeGymaeJaeGynaudacaGLOaGaayzkaaaaaa@88E4@

As we pointed out at the beginning of this section, the difference between the two cases above comes from the difference in the interpretation of what the model is and what the hypothesis is. In fact, if one assumes that the posterior of the hidden variable Z^i′
 MathType@MTEF@5@5@+=feaafiart1ev1aaatCvAUfKttLearuWrP9MDH5MBPbIqV92AaeXatLxBI9gBaebbnrfifHhDYfgasaacH8akY=wiFfYdH8Gipec8Eeeu0xXdbba9frFj0=OqFfea0dXdd9vqai=hGuQ8kuc9pgc9s8qqaq=dirpe0xb9q8qiLsFr0=vr0=vr0dc8meaabaqaciaacaGaaeqabaqabeGadaaakeaacuWGAbGwgaqcamaaBaaaleaacuWGPbqAgaqbaaqabaaaaa@2F8C@ is a part of the unknown parameter *θ*_*i*'_, then the second case is a special case of the original ODP. Namely, in case (a), the alternative hypothesis of a single gene is dependent on the expression distributions of the two groups of samples, while the alternative hypothesis in case (b) is dependent not only on the expression distributions but also on the class labels of the samples. When we ignore the multiplicity of statistical testing, these two cases are identical because the hidden variable is uniquely determined, even probabilistically, from the estimated parameter of the single-gene model.

The model in case (b) may have a biological meaning. The hidden variable vector *Z*_*i *_can be regarded as an on/off (binary) pattern vector of gene *i *over the samples. Since some biology, such as gene regulatory factors, may be represented as characteristic distributions of binary pattern vectors, the ODP framework will be able to apply such characteristics by sharing the underlying biological information in multiple tests.

## Results

Strictly speaking, a gene is called active if the true mean expression levels are different in different conditions regardless of whether the difference is large or small. However, in real situations it is impossible in principle to know whether a gene is truly active or inactive because its definition must be based on a finite (and often small) number of samples. Note also that it is difficult to confirm ever from any biological experiments whether any gene is truly active or inactive in the strictest sense – it is only a matter of the degree of significance.

In order to compare gene selection criteria, one solid way to consider an artificial situation where expression levels of truly active and truly inactive genes are given by probabilistic sampling from known probability densities on gene expression levels. On the other hand, we should also conduct experiments on real data sets because true distributions of gene expression levels of active and inactive genes are not necessarily the same as artificial distributions. Since we can expect that when the amount of information corresponding to the number of samples increases, gene rankings based on any statistical significance scores become more stable, we prepared a provisional target based on a sufficiently large number of labeled data sets. Based on the provisional target, we compared some gene significance scores with each other by evaluating stabilities against loss of information corresponding to loss of the number of labeled samples in realistic situations.

### Unsupervised differential gene discovery in artificial data

First of all, we compare various unsupervised scores devised for the detection of significant genes in an artificial setting.

**Figure 1:**
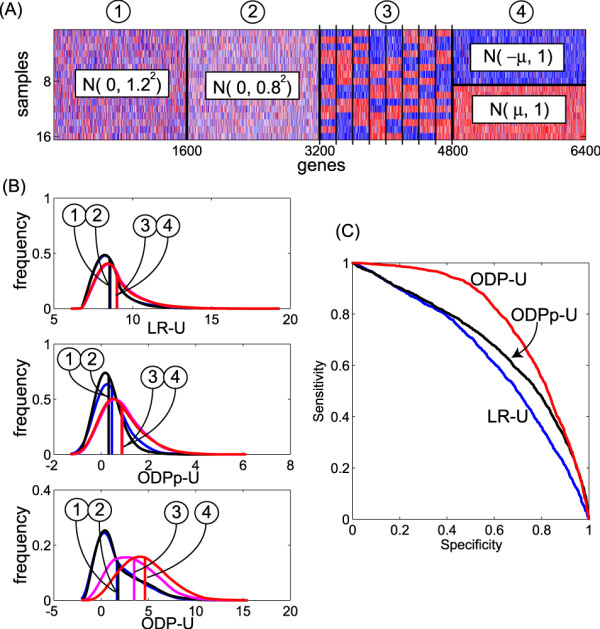
**Unsupervised extraction of significant genes**. (A) An artificial data set and its generative models. The artificial gene expression data set consists of 6,400 genes for 16 samples. The 6,400 genes are made up of 3,200 inactive ((1) and (2)) and 3,200 active ((3) and (4)) genes. Expressions of the inactive genes are generated from a normal distribution with mean 0 and variance 1.2^2 ^or 0.8^2 ^for gene group (1) or (2), respectively. Since the genes are generated from a single distribution regardless of the sample index, they are in fact inactive. Expressions of active genes are generated from a normal distribution with mean 1.0 or -1.0 for highly or lowly expressed genes in each sample, respectively, and a common variance 1.0. The expression pattern for each gene is different between groups (3) and (4): in group (3), there are eight subgroups of 200 genes and each subgroup has a high/low pattern different from the other subgroups. In group (4), 1,600 genes have the same high/low pattern. In (4), all the genes are assumed to reflect a common biology leading to similar expressions over all samples, and in (3) there are eight gene clusters, each of which reflects its own biology. (B) Histogram of three unsupervised significance scores: LR-U, ODPp-U, and ODP-U, which are separately described for the four gene groups. Horizontal axes are shown in log-scale. A vertical line denotes the mean of the distribution. (C) ROC (receiver operating characteristic) curves of active gene detection generated by changing the threshold for each score; the horizontal and vertical axes denote specificity, (true negative/(true negative + false positive)), and sensitivity, (true positive/(true positive + false negative)), respectively.

Figure 1A shows the structure of an artificial data set consisting of the expression of 6,400 genes for 16 samples. These 6,400 genes are made up of four groups, each of which includes 1,600 genes: groups (1) and (2) are inactive, and groups (3) and (4) are active, where the noise variance in group (1) is larger than in (2), and active genes are "on" in different samples of groups (3) and (4). The artificial gene expression levels are made from random samplings of normal distributions with certain means and variances, because logarithms of the gene expression ratios are known to be approximately normally distributed [10]. We compared the three scores listed below:

• LR-U, a gene-wise likelihood ratio, which is independent of the other genes;

• ODPp-U, an ODP based on the case (a) model, which shares the estimation of model parameters; and,

• ODP-U, an ODP based on the case (b) model, which shares the estimation of both model parameters and hidden variables.

Figures 1B and 1C show the results. In Fig. 1C, we can see ODP-U outperformed the others, i.e., it achieved the best sensitivity for each specificity, while LR-U was the worst. In Fig. 1B, we can see that the LR-U score is insensitive to the difference between the groups (1) and (2), or between (3) and (4). On the other hand, the score ODPp-U behaves differently between (1) and (2), i.e., the genes in group (1), which exhibit expressions with larger variance, tend to be evaluated as more significant because a larger variance likely causes a high chance of detecting active (possibly false) genes. However, the ODPp-U was insensitive to the difference between (3) and (4), which have the same expression distribution. The principal characteristic of the ODP-U score is that it extracted a larger number of active genes in group (4), suggesting an advantage of ODP-U. Since the 1,600 genes in group (4) have an identical pattern of true hidden variables, the commonality boosted the significance scores of genes in the same group. In group (3), the number of genes sharing the true hidden variables was 200, which led to weaker boosting of the significance scores than in group (4).

These results show that sharing the estimation of both the parameter and the hidden variable is effective in gene discovery when they have some structures that can be imprinted by making multiple tests cooperative. Also, we found that the more genes there are in the same group, the more effective is the sharing of the hidden variable estimation.

### Semi-supervised differential gene discovery on artificial data

Next, we consider the semi-supervised case. In the 16 samples, we assumed eight samples (index: 1,2,...,8) to have the true class label 1 and the other eight samples (index: 9,10,...,16) to have the class label 2. For each class, five samples were correctly labeled but the other sample labels were unknown. Note that the group (3) genes were assumed to be inactive in this case, because the sample labels represented by these genes (Fig. 1A) are not correlated with the labels of interest here. We compared the four scores listed below:

• SAM, SAM statistics;

• LR-S, likelihood ratio score;

• ODP-S, an ODP using only the samples with labels; and,

• ODP-SS, an ODP using all samples with the labels being known or unknown.

The three supervised scores, SAM, LR-S, and ODP-S, used the ten samples with labels to calculate their gene scores, while ODP-SS used, in addition, the remaining six unlabelled samples to estimate the unknowns in the hypothesis models.

**Figure 2:**
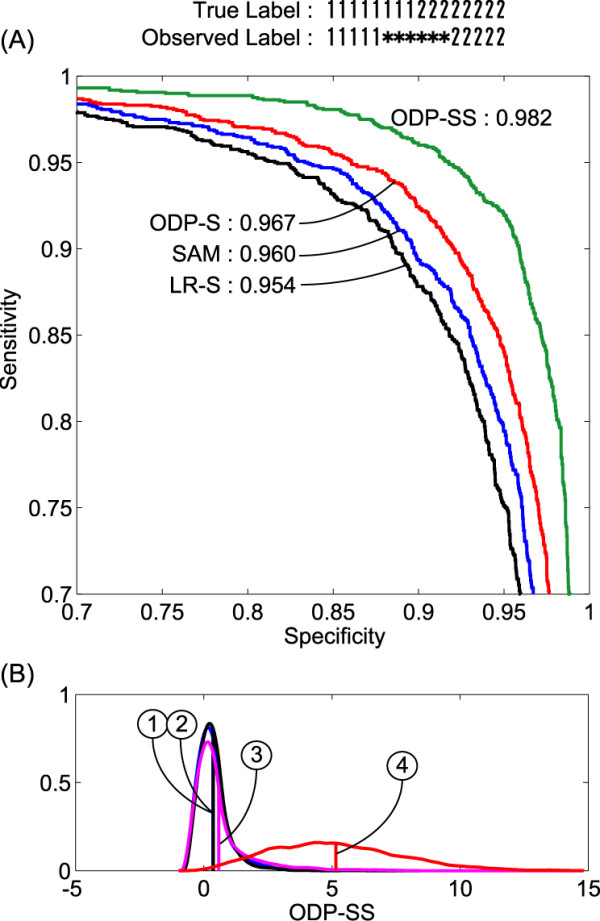
**Semi-supervised extraction of significant genes from the same artificial data shown in Fig. 1(A)**. The true labels of the 16 samples are 1 for the earlier eight samples and 2 for the rest, but three samples of both classes are assumed to be unknown (labeled as * in the figure title). This experiment evaluates how many genes in the truly active 1,600 genes of group (4) can be detected by four significance scores: LR-S, SAM, ODP-S, and ODP-SS. Note that the genes in group (3) are regarded as inactive in this experiment because the high/low patterns are different from the true label pattern. Therefore, there are 4,800 inactive genes (groups (1), (2), and (3)) and 1,600 active ones (group (4)). (A) ROC curves of the four scores. The number for each score denotes the AUC (area under the curve) values. (B) Distributions of the ODP-SS scores for the four gene groups, (1), (2), (3), and (4). Horizontal axis is shown in log-scale.

The ROC curves in Fig. 2A illustrate that ODP-SS achieved the best specificity for each sensitivity. Although SAM showed a better detectability than the conventional likelihood ratio, ODP-S exhibited far better specificity for each sensitivity. Figure 2B shows the distributions of ODP-SS for the four gene groups, (1), (2), (3), and (4). The active genes in group (4) were evaluated as clearly more significant than in the other groups by our ODP-SS score. However, the score distribution of gene group (3) is interesting. Although the distribution of expressions of inactive group (3) is identical to that of active group (4), the score distribution of group (3) is quite different from (4) but similar to those of (1) and (2) instead, which are also inactive with respect to the biology represented as the true label pattern. Even when the labeling includes uncertainty, the information of the labels affected the significance of the genes, which is stronger than the information of the expression distribution.

### Application to real data

In this section, we demonstrate the stability of the proposed semi-supervised and unsupervised scores based on two real data sets.

#### Prostate data set

The Prostate data set [11] consists of 52 prostate tumor samples and 50 normal samples, each of which was measured by origonucleotide microarrays (U95Av2 arrays, Affymetrix). Significant changes of expression levels were obtained at 10,509 probes out of approximately 12,600 genes and ESTs. In the original paper, signal-to-noise ratio is used to detect significant genes, and 317 genes with higher expression in the tumor samples and 139 genes with higher expression in normal prostate samples, i.e., 456 genes in total, were called significant based on a criterion, *p *< 0.001.

**Figure 3:**
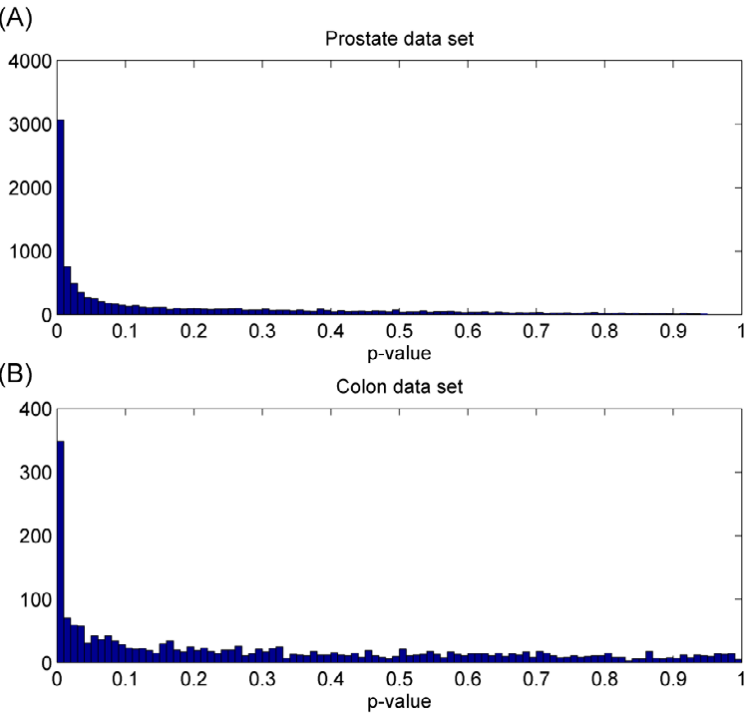
**P-value histograms for the two real data sets**. P-value histograms of all genes for the Prostate data set (A) and the Colon data set (B). Horizontal axes denote P-values calculated by EDGE [8] which is an application software based on the ODP-S score discussed in the current paper.

To overview of the data set, we consult the application software EDGE [8], which is based on an original implementation of ODP-S score and includes a procedure to estimate FDR. Figure 3A show the resultant histograms of p-values for the Prostate data set. From the p-value distribution, FDR is estimated for any threshold of the significance score, which indicates the proportion of the genes that are estimated as differential. When the top 1%, 10%, and 50% genes classified as significant, the estimated FDRs were 2.8 × 10^-6^, 0.00043, and 0.0105, respectively. For reference, the number of significant genes for the same threshold *p *< 0.001 obtained by EDGE, was 1293; this number is larger than the total number based on signal-to-noise ratio above, indicating higher detectability by the ODP.

#### Colon data set

The Colon cancer data set [12] consists of 40 colorectal tumor samples and 22 normal samples, each of which was measured by origonucleotide microarrays (Hum6000 arrays, Affymetrix). They selected 2,000 genes, from about 3,200 human cDNAs and 3,400 ESTs, which exhibited the greatest minimal intensity across the 62 samples. By applying EDGE to this data set (Figure 3B), when the top 1%, 10%, and 50% genes are classified as significant, the estimated FDRs were 0.00025, 0.0095, and 0.22. That indicates less proportion of genes in the Colon data set are significant than in the Prostate data set.

#### Experiment settings and results

We randomly masked sample label information except for 4, 6, 8, *N*/4, *N*/3, or *N*/2 samples, where the numbers of positive and negative labels in the non-masked samples are set to be same. Supervised scores SAM and ODP-S, and semi-supervised score ODP-SS are calculated based on the gene expressions and the non-masked labels, and corresponding significance rankings of all genes are also calculated. The relevance of these rankings is evaluated by concordance to the most reliable ranking based on the ODP-S score calculated by using the complete data without masked labels; i.e., this ranking is used as a provisionally true ranking.

Spearman's rank correlation, a well known criterion for evaluating the correlation between two different rankings, is used for evaluating concordance between two rankings:

RS(A,A′)=1−6N3−N∑i=1N(Ai−A′i)2,     (16)
 MathType@MTEF@5@5@+=feaafiart1ev1aaatCvAUfKttLearuWrP9MDH5MBPbIqV92AaeXatLxBI9gBaebbnrfifHhDYfgasaacH8akY=wiFfYdH8Gipec8Eeeu0xXdbba9frFj0=OqFfea0dXdd9vqai=hGuQ8kuc9pgc9s8qqaq=dirpe0xb9q8qiLsFr0=vr0=vr0dc8meaabaqaciaacaGaaeqabaqabeGadaaakeaacqWGsbGudaWgaaWcbaGaee4uamfabeaakiabcIcaOiabdgeabjabcYcaSiqbdgeabzaafaGaeiykaKIaeyypa0JaeGymaeJaeyOeI0YaaSaaaeaacqaI2aGnaeaacqWGobGtdaahaaWcbeqaaiabiodaZaaakiabgkHiTiabd6eaobaadaaeWbqaaiabcIcaOiabdgeabnaaBaaaleaacqWGPbqAaeqaaOGaeyOeI0IafmyqaeKbauaadaWgaaWcbaGaemyAaKgabeaakiabcMcaPmaaCaaaleqabaGaeGOmaidaaaqaaiabdMgaPjabg2da9iabigdaXaqaaiabd6eaobqdcqGHris5aOGaeiilaWIaaCzcaiaaxMaadaqadiqaaiabigdaXiabiAda2aGaayjkaiaawMcaaaaa@5191@

where *A*_*i *_and A′i
 MathType@MTEF@5@5@+=feaafiart1ev1aaatCvAUfKttLearuWrP9MDH5MBPbIqV92AaeXatLxBI9gBaebbnrfifHhDYfgasaacH8akY=wiFfYdH8Gipec8Eeeu0xXdbba9frFj0=OqFfea0dXdd9vqai=hGuQ8kuc9pgc9s8qqaq=dirpe0xb9q8qiLsFr0=vr0=vr0dc8meaabaqaciaacaGaaeqabaqabeGadaaakeaacuWGbbqqgaqbamaaBaaaleaacqWGPbqAaeqaaaaa@2F4A@ are the rankings of gene *i *according to two criteria *A *and *A'*, respectively. However, when we are interested in knowing whether each gene is top-ranked or not, and a detailed ranking of lower-ranked genes is not very important, the Spearman's rank correlation, *R*_S_, seems inappropriate because it is equally sensitive to differences of rankings both in top-ranked and bottom-ranked genes. We also considered another criteria. Area under the ROC curve, to detect the top *α*% genes, in short, AUC*α *is a criterion quite similar to the one we used in the previous simulation study except that true active genes are provisionally defined as the top *α*% genes of the provisional target ranking, ODP-S. We used *α *= 1 and 10 in the following experiment. The AUC*α *criterion can be used to evaluate the detectability of provisional genes from the objective ranking without determining the threshold of the objective ranking to define the significance or insignificance of genes. These two criteria are -1 ≤ *R*_*S *_≤ 1 and 0 ≤ AUC*α *≤ 1, and the chance levels for random rankings are 0 for the Spearman's criterion *R*_*S *_and 0.5 for the AUC*α *.

**Figure 4:**
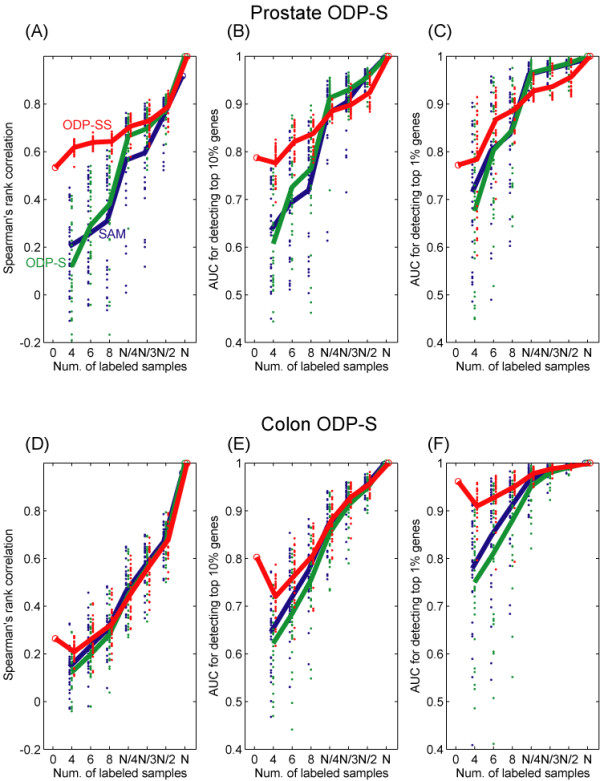
**Demonstration of semi-supervised score based on the real data sets**. Four comparisons based on Spearman's rank correlation ((A) and (D)), AUC 10% ((B) and (E)), and AUC1% ((C) and (F)). for the Prostate data set ((A), (B), and (C)), and for the Colon data set ((D), (E), and (F)). In each of the panels (A)-(F), the horizontal axis denotes the conditions, i.e., the numbers *K *of observed labels, where *N *is the total number of labeled samples in the original data sets. Blue, green, and red dots denote the compared scores, SAM, ODP-S, and ODP-SS, respectively, calculated based on 30 trials of random selection of the masked labels for each condition. Means of the 30 trials for each condition are plotted with lines in the colors of the corresponding dots.

Figure 4 shows the results of the three comparisons based on Spearman's rank correlation ((A) and (D)), AUC10% ((B) and (E)), AUC1% ((C) and (F)), for the Prostate data set ((A), (B), and (C)), and for the Colon data set ((D), (E), and (F)). In each of the panels (A)-(F), the horizontal axis denotes the conditions, i.e., the number *K *of observed labels, where *N *is the total number of labeled samples in the original data sets. Blue, green, and red dots denote the compared scores, SAM, ODP-S, and ODP-SS, respectively, calculated based on 30 trials of random selection of the masked labels for each condition. Means of the 30 trials for each condition are plotted with real lines in the same color as the corresponding dots. Note that the ODP-SS (denoted by red) at *K *= 0 is equivalent to the unsupervised score ODP-U, and the ODP-SS at *K *= *N *is equivalent to the supervised score ODP-S.

In the case of *K *= *N*, we confirmed that SAM and ODP-S showed very similar rankings from the results in which all of the three concordance criteria become nearly 1.0, which indicate the stability of the provisional target, i.e., ODP-S ranking on *K *= *N*. We performed a further experiment based on another provisional target, ranking of SAM on *K *= *N*, and we obtained almost identical results (not shown). For all rankings, all sample data sets, and all concordance criteria, we confirmed that concordance between any ranking to the provisional target tends to be lower, and their variances tend to be larger, for smaller numbers of labeled samples *K*.

Based on the Spearman's rank correlation to the provisional answer for the Prostate data set, Fig. 4A, the proposed semi-supervised ranking of genes, ODP-SS, showed better concordance than the rankings of SAM and of ODP-S for the cases with relatively small numbers of labeled samples *K*. ODP-SS at *K *= 0, i.e. the unsupervised ranking ODP-U, shows also moderately better concordance, which exceeds that of SAM and ODP-S at *K *≤ 8. These results indicate that unlabeled samples can improve the gene selection accuracy.

For the Colon data set, on the other hand, the Spearman's rank correlation to the provisional answer (D) did not display any prominent difference between any two rankings in the three objectives. Other criteria, AUC10% (E) and AUC1% (F), however, showed prominent the superiority of ODP-SS or ODP-U in cases when the number of labeled samples was smaller *K *≤ 8, which also indicates possible improvement. It is interesting that ODP-SS, based on a limited number of labeled samples, performed far worse than ODP-U which did not use any labels for the Colon data set. Furthermore, ODP-SS performed slightly below the supervised scores of ODP-S and SAM for the cases where the number of labeled data *K *were large for the Prostate data set. One possible reason for this is labeling error or its equivalent process. Labeling error is the result of confusion between tumor and normal samples that could not be trusted for inclusion in the original data set. However, in its equivalent process there may exist, some true active genes that more closely correspond to another binary label of samples than the tumor/non-tumor label. If the other label correlates closely enough with the original label, the detected set of significant genes will be almost the same between the scores based on the two labels. The degree of significance, however, may change depending on the selection of supervised or semi-supervised scores, because supervised scores rely heavily on the sample labels whereas semi-supervised scores rely greatly on the shapes of distributions. These possible effects of labeling errors should be considered further in future works.

We did not find any prominent difference between the supervised scores of SAM and ODP-S in these cases. That is, the two scores were almost equivalent with respect to the ability to detect a small number of top-ranked genes, and a small difference existed only in rankings among the top genes.

## Discussion

Discriminant analysis is another important aspect, different from differential gene detection, for selecting a small subset of important genes from microarray experiments. In discriminant analysis, a set of genes is determined to optimize the accuracy of sample prediction based on a certain method, such as using a support vector machine (SVM). As a typical example, the recursive feature elimination (RFE) method is considered to be one relevant way of optimizing the performance of SVMs [13]. In this case, however, the selected set of genes is not very meaningful biologically, because each one of the selected genes is not necessarily more important than the others, and no particular one of the remaining genes has enough reason to be ignored from a biological perspective. Differential gene detection is sometimes used for discriminant analysis. For example, the weighted vote method [14], and shrunken centroid [15] use a certain number of top-ranked genes ordered by classical criteria to evaluate differential genes, where only the number of top-ranked genes are determined based on sample discrimination accuracy with cross-validation or other devices. In short, although gene ranking based on statistical significance is useful for discriminant analysis, gene sets determined for improving discriminant analysis cannot be used to represent the significance of any single gene.

Semi-supervised analysis denotes the cases where unlabeled samples are used along with labeled samples. There have been many studies conducted on semi-supervised learning to improve discriminant accuracy by incorporating it with unlabeled samples from the general viewpoint of machine learning, ex. [16]. In addition, there is an application of semi-supervised learning to protein classification [17]. However, to the best of our knowledge there are no studies on semi-supervised detection of differential genes. There is, however, one on unsupervised gene selection based on entropy [18].

Bair and Tibshirani (2004) [19] proposed interesting procedures for sample discrimination by first selecting some significant genes using a certain supervised score, followed by a process that uses a certain unsupervised analysis, such as PCA or K-means clustering. They called their procedures "semi-supervised methods." Our study on semi-supervised detection of differential genes has no direct relationship to their "semi-supervised methods" because, from the viewpoint of machine learning terminology we adopt, their methods are actually "supervised" discriminant analysis rather than "semi-supervised." Their "supervised" methods, however, seem very good ways to avoid over-fitting to sample vectors when the number of samples is small, and the vectors are noisy and high-dimensional. Moreover, they will be further improved by using better differential scores of genes, such as by using our "semi-supervised" gene ranking incorporating many additional unlabeled samples, if available.

There had been many problem-specific devices proposed for modeling various effects of noise in microarray studies. Parametric models on noise in microarrays [20,21] or non-parametric treatment [22] improved the null and alternative distribution of microarray measurement. Bayesian hierarchical modeling can include various factors in a united manner, and there have also been some improvements in recent years, [23-25]. In the current study, we used the simplest normal mixture model for semi-supervised gene discovery, although the various devices mentioned above may further improve the accuracy in future works. In general, a differential gene-detection problem consists of two steps: ranking the genes according to the significance score derived for a given set of null and alternative hypotheses, and deciding the threshold for determining whether a gene is significant/insignificant based on the estimated false discovery rate (FDR) or other criteria. In this paper, we showed that the gene-ranking step can be improved by combining a latent variable model and the ODP framework, particularly in unsupervised and semi-supervised cases. We also showed that sharing the estimation of hidden variables is effective in the ODP framework for dealing with multiple testing.

Although Storey et al. [6,7] estimated an FDR based on ODP-S by using permutation of the class labels, we did not consider FDR in this paper. In an unsupervised case, class labels are missing and their permutation is impossible to perform, and in a semi-supervised case it is also difficult to consistently perform the permutation of labels. ODP requires a computational cost of *O*(*N*^2^), where *N *is the number of genes, and FDR estimation employing permutation would require the cost of a certain number of times (not small), as many as that for the ODP.

The need for better ranking never declines even when FDR estimation is not available. Gene ranking is typically required as an essential process for selecting candidate genes to reduce the cost of experiments in order to examine whether the top-ranked genes can be targets for medical treatment or can be as possible diagnosis markers. Therefore, ranking performance vital regardless of the existence of a way to determine the threshold by considering an FDR.

In this study, we demonstrated an improvement in gene-ranking estimation in semi-supervised and unsupervised cases for artificial and real data sets. We have shown that the stability of gene-ranking for small number of labeled samples can be drastically enhanced by incorporating unlabeled samples in a semi-supervised manner. On the other hand, for a sufficient number of labeled samples, the difference between the supervised and semi-supervised rankings was not prominent. Consequently, the optimal situation where the semi-supervised ranking of genes will exhibit prominent performance is in cases where the number of labeled samples is small whereas the number of unlabeled samples is large. There will be such a typical semi-supervised situation because sometimes it is not very difficult to measure a lot of samples by means of cheap microarrays, while to apply a label to each sample still incurs a large amount of time, money, and other miscellaneous costs. For example, to apply dead/alive in clinical applications may take several years to follow up.

Although the unsupervised score also exhibited fairly good concordance to the provisional supervised ranking corresponding to the difference between normal and tumor, it will not necessarily behave similarly in other situations corresponding, for example, to the difference between different types of tumors because the unsupervised score may pick up any genes that actively correspond to any differences. This characteristic of the unsupervised score was shown in the experiment using artificial data, where many genes in group (3), whose distributions have multimodality, were picked up even though they had no relationship to the main objective normal/tumor label. Consequently, the unsupervised score can effectively be used as an alternative way of filtering insignificant genes out in the pre-processing of gene expression analyses.

Applying various latent variable models such as a mixture of the t-distribution model, Cox's proportional hazards model, and so on, may lead to useful gene ranking within our framework, and such an application will be our future work.

## Conclusion

In general, sharing commonarities of hypothetical models over multiple tests has been thought as an important key of multiple testing problem. And, the ODP framework [6,7] had proposed a theoretically optimal way of sharing hypothetical models by sharing likelihood function based on maximum likelihood (ML) parameters.

We, in this paper, found that application of the ODP to latent variable models generally has two different possibilities, namely, the estimated hidden variables for other genes are shared or not. As an application of the ODP to latent variable models, we proposed new unsupervised and semi-supervised significance scores of genes based on a simple latent variable model, normal mixture model.

The simulation results indicated that the ODP framework is effective for hypotheses including latent variables and is further improved by sharing the estimations of hidden variables over multiple tests. The real data experiments showed that incorporation of the unlabeled samples by using the proposed unsupervised or semi-supervised score can improve the detectability of active genes.

## Authors' contributions

SO: Conducted research, designed study, designed artificial and real data simulation, wrote manuscript. SI: Wrote manuscript. Both authors read and approved the final manuscript.
